# Toll-Like Receptor 4 Limits Transmission of *Bordetella bronchiseptica*

**DOI:** 10.1371/journal.pone.0085229

**Published:** 2014-01-30

**Authors:** Olivier Rolin, Will Smallridge, Michael Henry, Laura Goodfield, David Place, Eric T. Harvill

**Affiliations:** 1 Department of Veterinary and Biomedical Sciences, The Pennsylvania State University, University Park, Pennsylvania, United States of America; 2 Graduate Program in Immunology and Infectious Disease, The Pennsylvania State University, University Park, Pennsylvania, United States of America; Universidad Nacional de La Plata., Argentina

## Abstract

Transmission of pathogens has been notoriously difficult to study under laboratory conditions leaving knowledge gaps regarding how bacterial factors and host immune components affect the spread of infections between hosts. We describe the development of a mouse model of transmission of a natural pathogen, *Bordetella bronchiseptica*, and its use to assess the impact of host immune functions. Although *B. bronchiseptica* transmits poorly between wild-type mice and mice lacking other immune components, it transmits efficiently between mice deficient in Toll-Like Receptor 4 (TLR4). TLR4-mutant mice were more susceptible to initial colonization, and poorly controlled pathogen growth and shedding. Heavy neutrophil infiltration distinguished TLR4-deficient responses, and neutrophil depletion did not affect respiratory CFU load, but decreased bacterial shedding. The effect of TLR4 response on transmission may explain the extensive variation in TLR4 agonist potency observed among closely related subspecies of *Bordetella*. This transmission model will enable mechanistic studies of how pathogens spread from one host to another, the defining feature of infectious disease.

## Introduction

Eliminating the transmission of infectious organisms is a primary goal of public health measures, yet laboratory studies that examine transmission remain sparse [1]–[6]. The paucity of transmission studies in animals may be largely due to the poor infectiousness of human pathogens in laboratory animals such as mice, rats and rabbits [7]. Infection of laboratory animals with human respiratory pathogens often requires large inocula that may swamp the system and bypass key steps required to establish natural infections [8]. The respiratory bacterium *Bordetella bronchiseptica* is a suitable model pathogen for examining transmission in the laboratory because it is highly infectious in mice and experimental infections can be carried out with low doses, simulating naturally transmitted infections. In two prior studies transmission of *B. bronchiseptica* has been observed in swine [5], [6], but not in the mouse model that is more amenable to experimental manipulation of the many aspects of host response. Although *B. bronchiseptica* is rarely found in humans it is closely related to the evolutionary progenitor of *Bordetella pertussis* and *Bordetella parapertussis*, the causative agents of whooping cough in humans. Many virulence factors are highly conserved between these species, thus the study of *B. bronchiseptica* transmission in mice can provide insights into processes underlying the transmission of *Bordetella* among humans.

Previous studies in mice have produced network models for immune control of *Bordetella* infections [9], [10]. Innate immune receptors and cytokines that regulate the inflammatory cascade such as toll like receptor 4 (TLR4) [11], tumor necrosis factor-alpha TNFα [12] and interleukin-1 receptor (IL1R) [13] are necessary for host survival when mice are challenged with large doses of *B. bronchiseptica*. Adaptive immune components, such as serum antibodies, B-cells [14], T-cells [15] and T-helper-1 cytokine interferon gamma (IFNγ) [16] are required to ultimately clear infectious organisms from the lower respiratory tract and reduce colonization in the upper respiratory tract. How elements of immunity affect transmission has not previously been defined for lack of a suitable experimental system.

Though low doses of *B. bronchiseptica* efficiently colonize the mouse respiratory tract, transmission of *B. bronchiseptica* between wild-type mice is rarely observed [17]. For transmission to take place, bacteria must shed from infected individuals and arrive in sufficient number into their niche in another host. The low frequency of transmission of *B. bronchiseptica* between wild-type mice suggests that mice either shed insufficiently or that they are highly resistant to colonization. In rabbits, the intensity and duration of shedding varied with inter-individual differences in bacterial load, serum antibody titers and cytokine responses, demonstrating that the nature and strength of immune responses affect the transmissibility of *B. bronchiseptica* [18]. Since immunity may restrict the transmission of *B. bronchiseptica*, we employed the tools available for mouse immunology to investigate how host immune components affect transmission.

We carried out infections with *B. bronchiseptica* in wild-type, antibody depleted, or genetically defined immunocompromised mice, and measured parameters that affect pathogen transmissibility: 1) experimental infectious dose 2) growth and persistence of bacteria within the host, 3) the number of organisms shed to the environment over time, and 4) the frequency of transmission from infected hosts to cohoused, naïve mice. Our results showed that innate immune activity regulated by the Toll-like Receptor 4 (TLR4) limits transmission of *B. bronchiseptica* between mice and has a strong influence on all measurable parameters associated with transmission. While adaptive immunity was not required to control transmission, TLR4 activity conferred resistance to initial colonization, restricted growth and persistence within the respiratory tract, and reduced shedding of *B. bronchiseptica* from the nares. Surprisingly, the absence of TLR4 signaling led to increased recruitment and polymorphonuclear cells (PMN) and the intensity of PMN recruitment affected shedding independently of bacterial numbers within the host. Together these results indicate that TLR4 regulation of innate immunity and inflammatory responses is central for controlling transmission of *B. bronchiseptica.*

## Results

### TLR4-dependent innate immune responses control transmission of *B. bronchiseptica*

**Figure 1:**
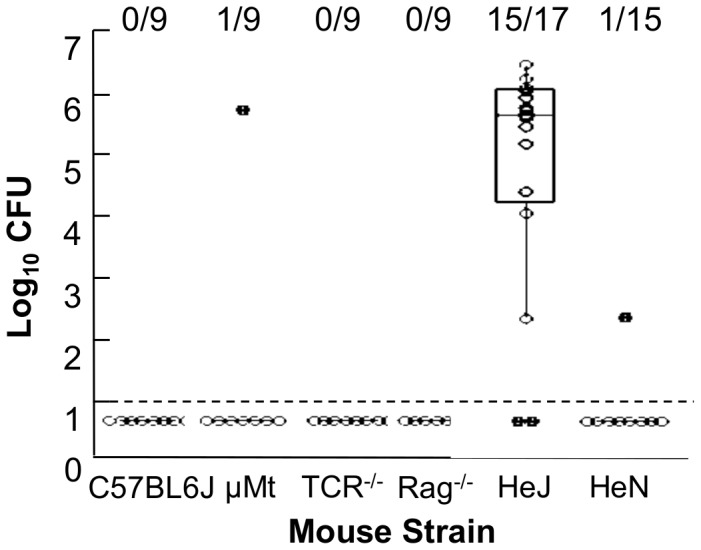
TLR4 is required to prevent transmission of *B. bronchiseptica* between mice. The number of CFU of *B. bronchiseptica* recovered from the nasal cavity of HeJ, HeN, C57BL/6 , μMT, TCR^−/−^, or Rag^−/−^ secondary mice dissected after three weeks of exposure to an index case. Index cases were of the same genotype as secondary mice. Individual secondary mice are represented by dots. Box spans the interquartile range, and the median value is represented by a bar. The whiskers span the 1st and 4th quartile. The limit of detection is marked by a dashed line. The proportion of infected mice in each group is listed above the box and whisker plot. Figure represents the cumulative results of 2–3 independent experiments for each mouse genotype.

To determine whether specific components of immunity limit transmission of *B. bronchiseptica*, wild type or various knockout mice were inoculated with 100 CFU *B. bronchiseptica* (index mice) and immediately placed in cages with 2 or 3 naïve mice (exposed/secondary mice) of the same strain. Four weeks later all mice in the cage were dissected and the nasal tissue was cultured to determine whether *B. bronchiseptica* had been transmitted to secondary mice (Figure 1). B cell-deficient (μMt), T cell deficient (TCRβδ^−/−^) or combined B and T cell deficient (Rag^−/−^) mice were included in this experiment to examine the role of adaptive immunity in transmission. Following cohousing with an index mouse, zero of the nine wild-type C57BL6/J (C57) secondary mice had become infected by *B. bronchiseptica*. One transmission event was observed among eight B-cell deficient (μMT) mice and no transmission events were observed in mice lacking T cells (TCRβδ^−/−^) or lacking both B and T cells (Rag^−/−^) (eight mice each). The frequency of transmission among groups of μMT, TCRβδ^−/−^ and Rag^−/−^ mice (4.2%; 0–12%) (Mean; 95% confidence interval) was not significantly different from that observed in C57 mice.

Though adaptive immunity did not affect transmission of *B. bronchiseptica*, rapid initiation of innate immune responses may limit a pathogen's success during early stages of infection. Because TLR4 is central for rapid activation of innate immune responses upon high dose challenge with *B. bronchiseptica*, we examined transmission of *B. bronchiseptica* between TLR4-mutant C3HeJ (HeJ) mice [11], [12]. Fifteen of seventeen (88.2%; 74%–99%) HeJ secondary mice became colonized by *B. bronchiseptica* (Figure 1; Table 1), while only a single secondary mouse among the 15 strain-specific control wild-type C3HeN (HeN) (.066; 0–.195) was colonized, demonstrating that TLR4 inhibits transmission of *B. bronchiseptica* amongst these mice.

**Table 1:** Frequency of Transmission Events Between Mice by Genotype.

Index Case	Recipient	Frequency of Transmission	95% Confidence Interval
HeJ	HeJ	88%	74–99%
HeN	HeN	6.7%	0.0–19%
C57BL6J	C57BL6J	0.0%	NA
μMt	μMt	11%	4–18%
TCRβδ^−/−^	TCRβδ^−/−^	0.0%	NA
Rag^−/−^	Rag ^−/−^	0.0%	NA
HeJ	HeN	13%	1–26%
HeN	HeJ	53%	32–74%

The observed proportion 95% confidence interval for the frequency with which of secondary mice of secondary mice that became infected in the presence of an index mouse as a function of genotype.

### Genetic immunodeficiency does not affect resistance to colonization

**Figure 2:**
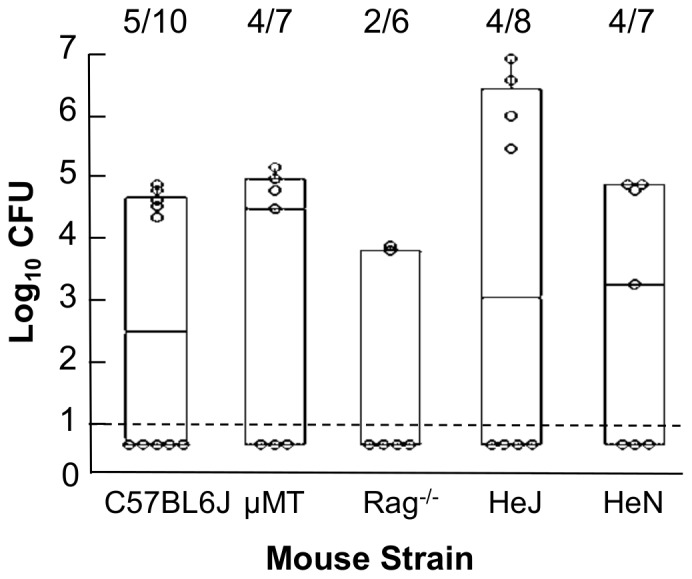
Immunodeficiency does not affect resistance to experimental infection with *B. bronchiseptica*. Mice were inoculated with 5*B. bronchiseptica* in 5 µl of PBS and dissected 7 days later. The quantity of *B. bronchiseptica* recovered from the nasal cavity of individual mice is represented by dots. Box spans the interquartile range with the line denoting the median value. Whiskers span the 1st and 4th quartile. The limit of detection is marked by a dashed line. The proportion of infected mice in each group is listed above the box and whisker plot. Figure represents the cumulative results of 2 independent experiments for each mouse genotype.

We attributed the high frequency of transmission between HeJ mice to either increased bacterial shedding by index mice or decreased resistance of secondary mice. To determine the effects of specific immune components on resistance to colonization we inoculated groups of six to eight C57, μMt, Rag^−/−^, HeN or HeJ mice with 5 CFU of *B. bronchiseptica* in 5 µl of PBS. Seven days following infection, quantitative culture of the nasal cavity showed no difference in the proportion of mice infected, suggesting that increased transmission between TLR4 deficient HeJ mice was not due to decreased resistance to colonization (Figure 2).

### Innate immunity limits growth of *B. bronchiseptica* and prevents spread to the lower respiratory tract

**Figure 3:**
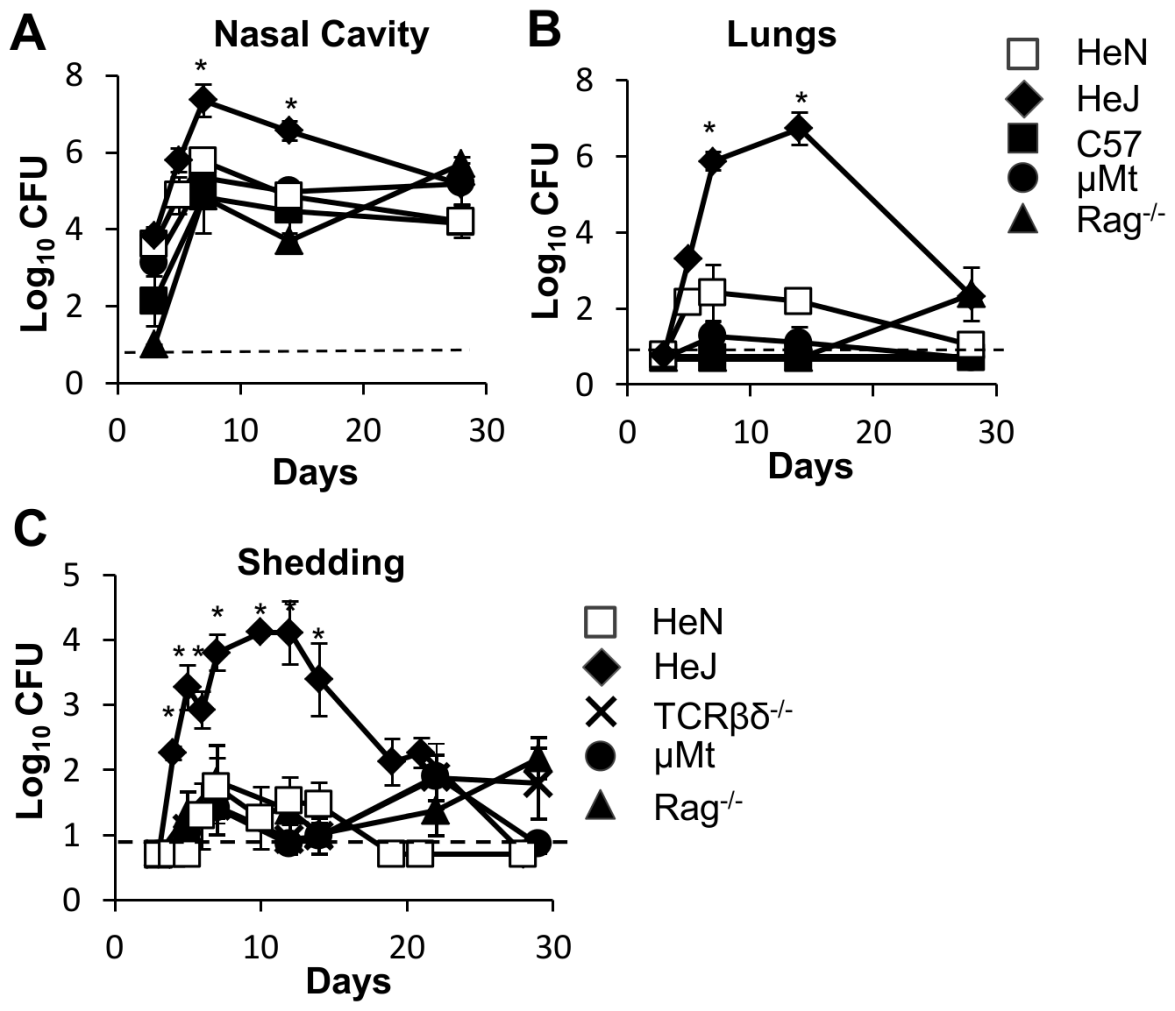
TLR4 is required to limit the growth of *B. bronchiseptica* within the respiratory tract. 3–4 mice per group were examined at 3, 7, 14, and 28 days after inoculation. The bacterial loads of *B. bronchiseptica* recovered from the (**A**) nasal cavity or (**B**) lungs of wild-type (dashed lines) HeN (white squares) or C57BL/6 (black squares), and immunodeficient mice (solid lines): HeJ (black diamonds), μMt (black circles) or Rag^−/−^ (black triangles). (**C**) Shedding was detected throughout the infectious course by quantitative culture of bacteria obtained in a ten second swab of the external nares. Symbols represent the mean log_10_ CFU of *B. bronchiseptica* (± SEM) Dashed line represents the limit of detection. Figure represents 1 of 2 independent iterations of this experiment.

Immune-mediated control of the pathogen load within the host may limit the numbers of bacteria communicated to their contacts. To determine the contribution of immune components to the control of bacterial numbers over time, within-host bacterial burden was measured at several intervals throughout a four week time course. We inoculated, C57BL6/J, μMT, Rag^−/−^, HeJ, and HeN with 100 CFU of *B. bronchiseptica*, and dissected 4 mice per group 3, 7, 14 and 28 days later (Figure 3). The peak of infection was detected 7 days post inoculation for all groups, and throughout the time course no significant difference in the number of CFU recovered from the nasal cavity was observed between WT mice and mice lacking adaptive immune components (Figure 3A). Likewise, colonization of the lungs was similar amongst these mice and never exceeded 10^3^ CFU at any time point (Figure 3B). However, in HeJ mice, the nasal colonization by *B. bronchiseptica* was significantly greater than in all other groups, (*P<*0.01), and at the peak of infection HeJ mice had approximately a 20-fold greater burden of *B. bronchiseptica* in the nasal cavity (*P<*0.05) than did WT mice (Figure 3A). The CFU of *B. bronchiseptica* recovered from the lungs of HeJ mice was also significantly greater than that recovered from WT, Rag^−/−^ and μMT mice (*P<*0.01), with more than 10^6^ CFU observed at day 7 and day 14 post innoculation.

### Innate immunity limits shedding intensity and duration

To determine whether a greater burden of within-host bacteria was associated with increased shedding of organisms from infected mice we assessed the bacterial load shed from the external nares of infected mice throughout the course of infection. We inoculated 100 CFU of *B. bronchiseptica* into μMT, TCRβδ^−/−^, Rag^−/−^ , HeJ, and HeN mice and swabbed their external nares periodically over a 28 day period (Figure 3C). Shedding was maximal at day 7 post-innoculation for C57BL6/J, μMT, TCRβδ^−/−^ and Rag^−/−^ hosts and no significant difference in shedding intensity was observed between these groups until 18 days post inoculation when shedding from WT mice fell below detectable levels. μMT, TCRβδ^−/−^ and Rag^−/−^ continued to shed 10–100 CFU from Day 7 after inoculation until the end of the timecourse. In contrast, over most of the time course shedding of *B. bronchiseptica* from HeJ mice was greater than that of HeN, μMT, TCRβδ^−/−^ or Rag^−/−^ mice (*P<*0.01). Approximately 10–1000-fold more CFU shed at each time point measured between day 5 and day 21 post-innoculation(*P<*0.01), indicating that TLR4 limits shedding of *B. bronchiseptica* from infected mice.

### More neutrophil recruitment is observed in TLR4 mutant mice

**Figure 4:**
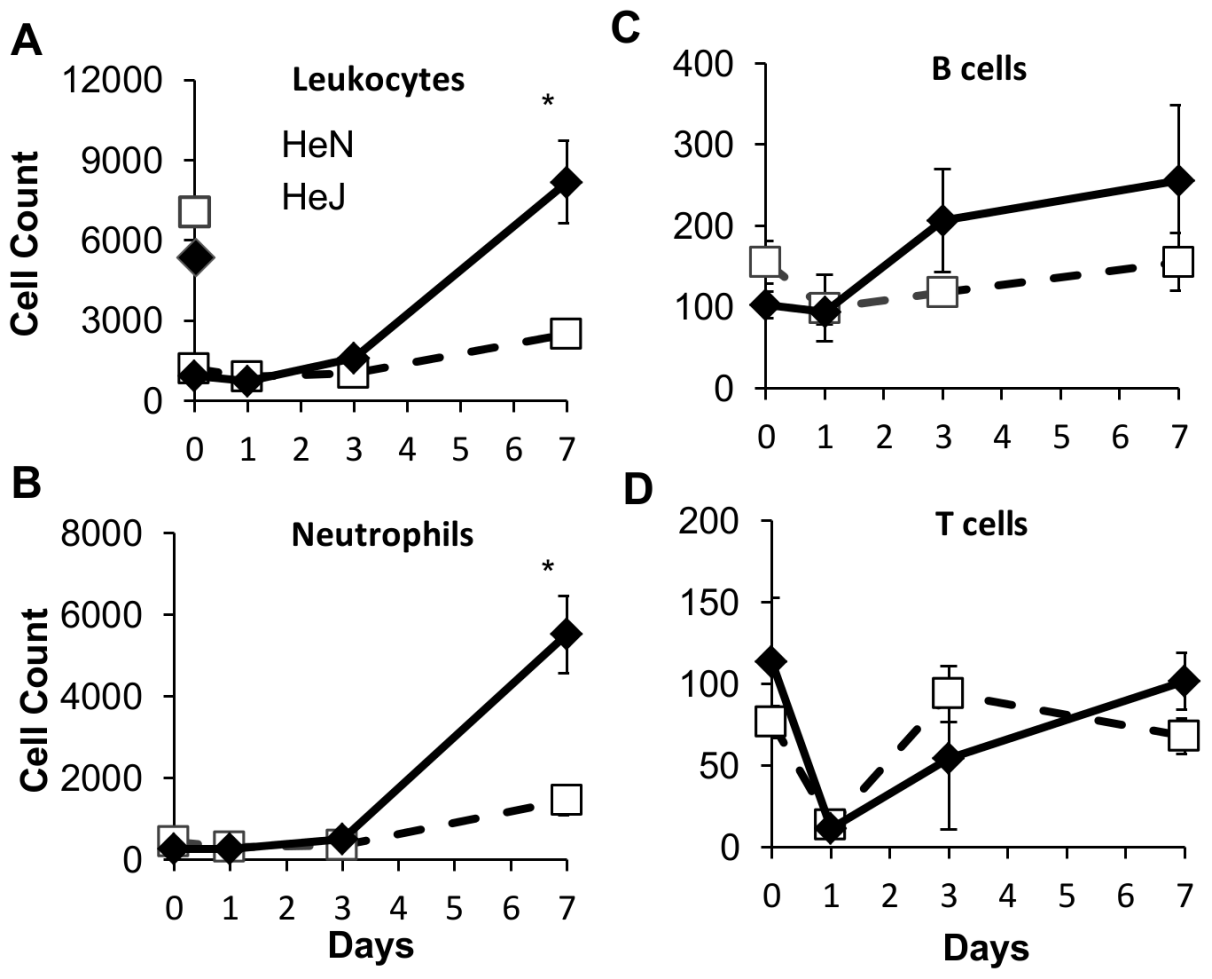
Intense leukocyte and neutrophil recruitment to the nasal cavity is observed in HeJ but not HeN mice. Symbols represent the mean ± standard error of flow cytometry-derived counts of white blood cell types: (**A**) Total leukocytes (CD45^+^), (**B**) neutrophils (CD11b^+^/Ly6G^+^), (**C**) macrophages (F4/80^+^/MHCII^+^), (**D**) NK cells (NK1.1^+^), (**E**) B cells (B220^+^/MHCII^+^), and (**F**) T cells (CD3^+^) recovered from the nasal cavity of mice immediately before (day 0) and 1, 3 and 7 days after inoculation of HeJ (black diamonds) or HeN (white squares) mice with 100 CFU of *B. bronchiseptica*. Experiments were carried out with 4 mice per group in 2 separate iterations.

Loss of TLR4 activity in HeJ mice may contribute to multiple sequential deficiencies in immune activity including decreased production of antimicrobial defenses by epithelia, delayed recruitment of innate immune leukocytes to the site of infection or compromised activation of adaptive immunity. To determine whether defects in the leukocyte response could account for poor control of infection and transmission in the absence of TLR4, we inoculated 100 CFU of *B. bronchiseptica* into groups of four HeJ or HeN mice. Mice were dissected one, three, and seven days after infection and the presence of various leukocyte subsets in the nasal cavity was determined by flow cytometry (Figures 4, S1 and S2). Over the first three days no significant changes in the total leukocyte population was observed when compared with naïve mice. Although the number of B or T cells present did not increase over baseline levels (Figure 4C, D), macrophages and NK cells increased more than four-fold over baseline levels (*P*<0.05) in both HeJ and HeN mice (Figure 4A, Sup2). By seven days post inoculation WT mice had a two-fold increase in total nasal leukocytes while HeJ mice had a six to eight fold increase, significantly more than HeN (*P*<0.05) (Figure 4A). Of the cell types analyzed, neutrophils (PMN) represented the most significant population and were the only subset that differed significantly between HeJ and WT mice. Relative to naïve mice, PMN counts increased two-fold in *B. bronchiseptica* infected WT mice (*P<*0.05), whereas a ten-fold increase was observed in HeJ mice (*P<*0.01) (Figure 4B).

**Figure 5:**
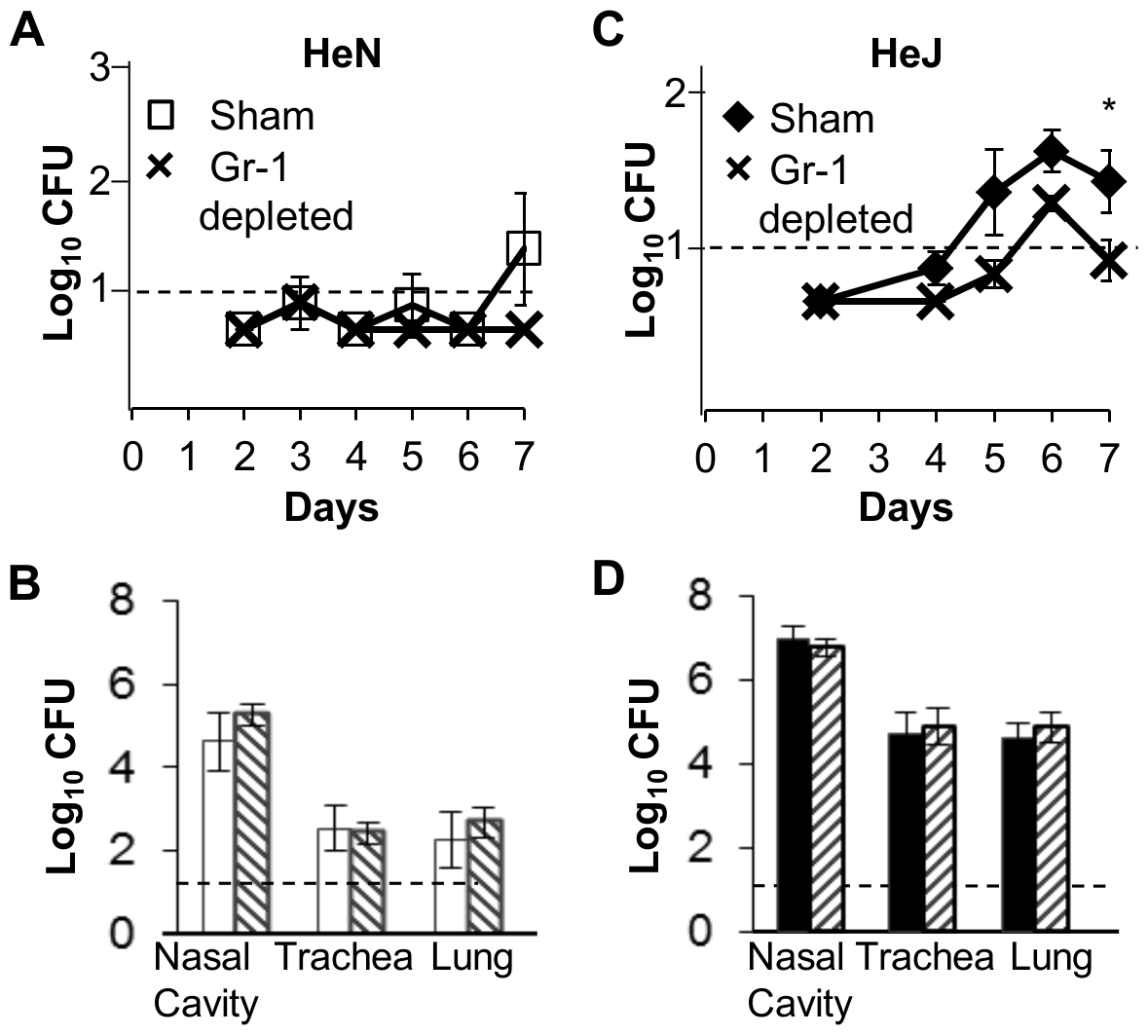
Depletion of neutrophils reduces shedding from HeJ mice and does not affect within host colonization. (**A**) HeN or (**C**) HeJ mice were given an intraperitoneal injection of 0.5 mg of either isotype control IgG2b (HeN white squares; HeJ black diamonds) or anti-GR1 (black crosses) antibodies prior to infection and every other day thereafter. Shedding was detected by quantitative culture of bacteria obtained in a ten second swab of the external nares. Symbols represent the mean log_10_ CFU of *B. bronchiseptica* ± the standard error. The number of CFU of *B. bronchiseptica* recovered from the nasal cavity, trachea, lung, liver, and spleen of (**B**) HeN or (**D**) HeJ mice treated with either isotype control IgG2b (HeN white bars; HeJ black bars) or anti-GR1 antibodies (striped bars) 7 days after inoculation. Bars represent the mean log_10_ CFU of *B. bronchiseptica* ± SEM. Dashed line represents the limit of detection. Experiments were carried out with 4 mice per group in 2 separate iterations.

Examining the shedding output of individuals as a function of the neutrophil recruitment response, we found that for both HeN and HeJ mice, individuals with the highest shedding output at 7 days post inoculation also had the highest neutrophil counts, and in general there was a strong positive correlation between neutrophil counts and shedding output (Figure S3). To determine whether PMN played an important role in the control of infection or whether they directly contributed to exacerbated infection and shedding, groups of four HeJ or HeN mice were depleted of PMN with anti-Gr1 antibodies given IP one day prior to infection, and every other day until seven days post infection. Shedding was monitored over time (Figure 5A) and mice were sacrificed seven days after inoculation to quantify the burden of bacterial colonization within the host (Figure 5B). No shedding was detected from HeN mice depleted of PMN while a small amount was detected from untreated controls. In Gr1-depleted HeJ mice shedding was slightly but significantly decreased over the seven day time course compared with control HeJ mice (*P<*0.05) (Figure 5B). There was no difference between bacterial numbers recovered from mice depleted of PMN compared with sham-treated control mice. Anti-Gr1 treatment successfully depleted nasal neutrophils up to seven days post inoculation (Figure S4).

When PMN depletion was carried out over 14 days shedding output was significantly higher in the Gr1-depleted mice than in controls, particularly from days 9 to 14 (*P<*0.05) post inoculaltion (Figure S5A). Analysis of blood samples taken from this cohort 7 days after initiating anti-Gr1 antibody therapy showed that PMN were effectively depleted at 7 days; however, by 14 days after inoculation PMN counts in anti-GR1 treated WT mice were approximately 10-fold higher (*P<*0.05) (Figure S5B). Shedding from HeJ mice depleted of PMN was significantly reduced over the time course (*P* = 0.01). Similarly to HeN mice, anti-GR1 treatments effectively depleted PMN in HeJ mice for 7 days, however at day 14 neutrophil counts in anti-GR1 treated and control HeJ mice were not different (Figure S5E). The burden of bacteria within the nasal cavity of either HeN or HeJ mice on day 14 after inoculation was not affected by treatment with anti-Gr 1 antibodies (Figure S5C, F).

### TLR4 confers increased resistance to colonization in mice exposed to infected hosts

**Figure 6:**
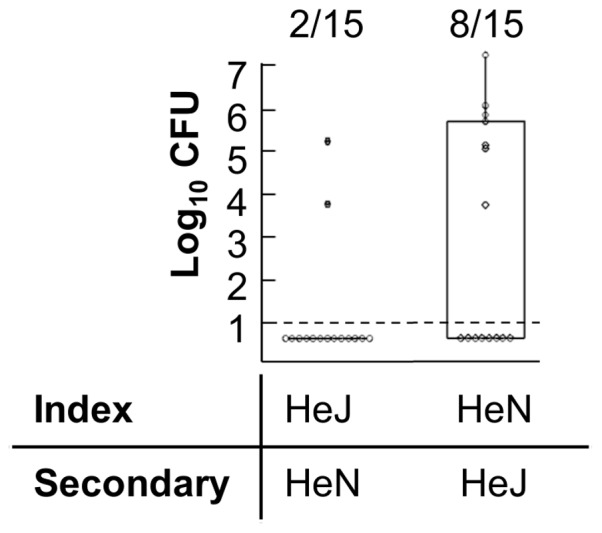
B. bronchiseptica recovered from secondary mouse nasal cavity. CFU of B. bronchiseptica were recovered from the nasal cavity of HeN secondary mice three weeks after exposure to a HeJ index case (left) or HeJ secondary mice four weeks after exposure to a HeN index case (right). Individual secondary mice are represented by dots. Box and spans the interquartile range, while the whiskers span the 1st and 4th quartile. The proportion of infected mice in each group is listed above the box and whisker plot. Dashed line represents the limit of detection. Figure represents cumulative findings of 2 iterations of the same experiment using 4–5 mice per group.

To determine whether increased shedding accounted for the difference in transmissibility of *B. bronchiseptica* in HeJ mice compared to WT HeN mice, 100 CFU of *B. bronchiseptica* was inoculated into HeJ mice or HeN mice. Infected HeN mice were placed in cages with HeJ secondary mice, or conversely infected HeJ mice were co-housed with HeN secondary mice (Figure 6). After 4 weeks of cohousing only 2 of 16 (0.125: 0.01–0.27) HeN secondary mice exposed to high shedding HeJ mice were colonized by *B. bronchiseptica*, whereas HeN index mice transmitted *B. bronchiseptica* to 8 of 15 (0.533: 0.31–0.65) HeJ secondary mice (Figure 6).

### Transmission to secondary mice becomes detectable by ten days after inoculation of index mice

**Figure 7:**
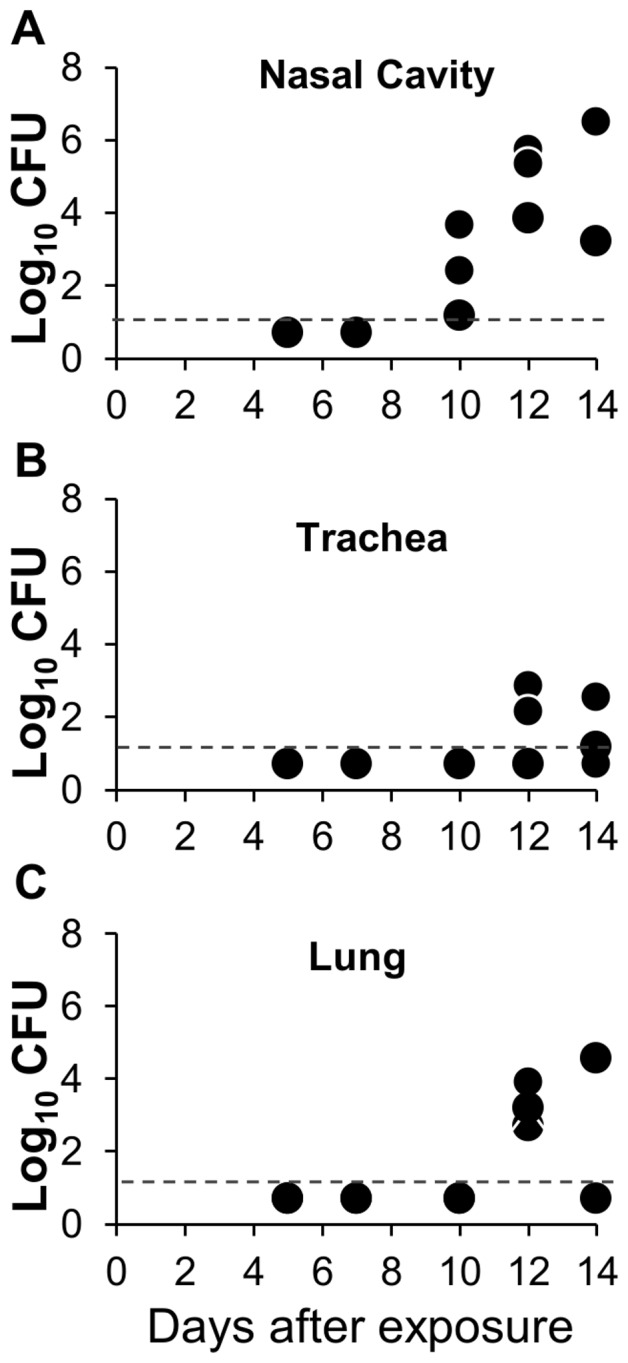
Naturally transmitted *B. bronchiseptica* infections begin in the nasal cavity before growing and spreading to the lower respiratory tract. Index HeJ mice were inoculated with 100(**A**) nasal cavity, (**B**) trachea, or (**C**) lungs of individual recipient HeJ mice after being housed with an index case for 5, 7, 10, 12, and 14 days. The dashed line indicates the limit of detection. Graph represents findings of a single experimental iteration.

Having demonstrated that *B. bronchiseptica* transmits efficiently between HeJ mice we, used this model to estimate at what point infection is detectable in secondary mice following exposure to index mice. HeJ index mice were inoculated with *B. bronchiseptica* and immediately co-housed with HeJ secondary mice. Groups of three secondary mice were sacrificed at two to three day intervals between day five and day fourteen after initial infection of index mice. *B. bronchiseptica* was not detected in secondary mice at five or seven days after cohousing, whereas all mice sacrificed at day ten or later had become infected with *B. bronchiseptica*. In mice sacrificed at day ten the burden of colonization ranged from 20 CFU to 2000 CFU in the nares, while no colonization of the trachea or lungs was detected at this time. Colonization burden in the nares of mice sacrificed at days twelve and fourteen was significantly greater than found on day 10 (Figure 7A) and lung and tracheal colonization could be detected at these time points (Figure 7B,C).

## Discussion

TLR4 signaling prevented transmission of *B. bronchiseptica* both by decreasing the intensity and duration of shedding from infected hosts while also contributing to the resistance of naïve mice exposed to infected hosts. Although TLR4 may affect both innate and adaptive immunity, the high shedding output of infected TLR4 deficient, HeJ mice compared with wild type mice was likely due to disruption of innate immune responses, since peak levels of shedding occurred relatively early in the infectious process and were not affected by major defects in adaptive immunity (Figure 4A,B). Intense shedding from HeJ mice corresponded with a high pathogen load within the host as well as intense PMN recruitment in these mice (Figure 4C). Because HeJ index mice transmitted more frequently (88%) than wild-type HeN index mice (53%) to HeJ secondary mice (Table 1), we concluded that increased shedding intensity contributed to transmission. The low frequency of transmission from high shedding HeJ index mice to HeN secondary mice (0.125) (Table 1) demonstrates that host resistance to initial colonization is the primary determinant of transmissibility of *B. bronchiseptica* among mice. We were surprised to find that colonization resistance was important in preventing transmission since inoculation with only 5 CFU was sufficient to infect more than 50% of all mice (Figure 2). This finding suggests that transmission by host-to-host contact has important mechanistic elements that cannot be entirely reproduced by experimental inoculation. Despite the limitations of inoculation, we observed that infections derived by contact with another host became detectable only after 7 days affirming that high shedding output by index mice facilitates transmission. Despite exposure to mice shedding thousands of organisms, our results find that relatively small quantities of bacteria are detected in the nares of secondary mice (20–2000) before the infection grows and spreads throughout the respiratory tract (Figure 7). This finding supports the use of low dose, low volume inoculation for better experimental approximation of natural infections with *B. bronchiseptica* in animals.

TLR4 may affect the transmission dynamics of multiple Gram-negative pathogens. Polymorphisms resulting in hypo-responsive TLR4 signaling are associated with susceptibility to Gram-negative sepsis as well as infections by *E. coli* and *Meningococcus* [19]. The ability of TLR4 signaling to prevent transmission may also drive the evolution of lipid-A structures that modulate their TLR4 agonist activity. *Yersinia pestis* produces a TLR4 agonist lipid-A at room temperature, however at 37 degrees *Yersinia pestis* produces a TLR4 antagonist lipid-A structure. As lack of TLR4 signaling may lead to impaired immune responses, evasion of TLR4 may correspond with its intense virulence when transmitting between humans as a respiratory pathogen and its reduced virulence when transmitted by ectothermic insect vectors [20], [21]. Similarly *Bordetella pertussis*, and *B. parapertussis*, which are weak agonists of human TLR4 compared with *B. bronchiseptica* [22], [23] cause more severe disease within their hosts and are among the most contagious pathogens circulating in the human population [24].

Although it is unclear how wild-type HeN mice achieve superior control of *B. bronchiseptica* compared with TLR4 deficient HeJ mice, heavy PMN infiltration in HeJ mice marked the chief difference in the leukocyte profiles between these mice (Figure 4D). Increased PMN recruitment during infections of TLR4 deficient mice by Gram-negative pathogens *Pseudomonas aeruginosa*, *Neisseria gonorrhea*, *Haemophilus influenzae* has been reported previously [25]–[27]. PMN from TLR4-mutant mice kill *N. gonorrhea* and *H. influenzae* less effectively than PMN from wild-type mice [26], [27] and either mutation in TLR4 or depletion of PMN from wild-type mice diminishes immune control of *H. influenzae* [28]. In our infection system, depletion of PMN from WT HeN mice did not affect bacterial load within the host, suggesting that PMN are not required to control *B. bronchiseptica* infection and that poor function of PMN does not account for the severity of infection in HeJ mice. Intense PMN recruitment in HeJ mice may be a result of poor control of infection, or perhaps is initiated by an alternative response pathway that is normally suppressed by TLR4. Higgins et al. demonstrated that during *B. pertussis* infections TLR4 protects mice from inflammatory immunopathology by induction of T regulatory cells suggesting that TLR4 not only directs antimicrobial defenses, but also inhibits overactive responses [29]. A recent paper by Gopinath et al. that studied shedding in a mouse model of *Salmonella typhi* infection demonstrated that individual mice within a cohort that were high shedding outliers (supershedders) had abberant neutrophil responses. Reciprocally supershedder mice had suppressed adaptiveTH1 responses [30]. When exaggerated neutrophil responses were induced ectopically in normal shedding mice with gm-csf the shedding was greatly enhanced as TH1 responses were compromised. The Gopinath model effectively corroborates the effect of neutrophils on shedding observed in our study; however the relatively good control of shedding in TCR^−/−^, μMt, and RAG^−/−^ mice suggest that compromised adaptive immunity does not explain high shedding in our model. We observed that depleting PMN decreased the shedding by HeJ mice (Figure 5C), whereas aberrant, large numbers of PMN present in the nasal cavity of HeN mice after 14 days of anti-Gr1 therapy corresponded with increased the shedding by HeN mice without affecting within host numbers (Figure S5a–c). Because shedding output may be modulated by experimentally induced alterations of the measured the neutrophil response, but is not affected by the absence of T or B cells, we suggest that PMN recruited to the site of infection may affect transmission through a primary effect on the shedding process.

We make the above claim with a measure of caution since the increased recruitment of neutrophils discovered in mice after 14 days of anit-Gr1 therapy was unexpected and we do not have data to determine at which time point between day 7 and 14 depletion begins to fail. We suspect that failure to deplete neutrophils at 14 days was due to a host antibody response neutralizing the Gr-1 antibody, but this has not been addressed experimentally. It is also unclear why there is a 10-fold increase in the neutrophil response after failure of depletion therapy. We suspect that transient neutropenia may cause a compensatory increase in bone marrow production of PMN.

Inflammatory responses enable hosts to control infections [31] by production of broad spectrum antimicrobial enzymes, peptides and reactive oxygen species [32]. Inflammation also enhances T cell priming and recruitment of adaptive immune lymphocytes [33] thus it has been difficult to reconcile why certain *Bordetella* virulence factors promote inflammatory pathology in the lungs [34]. In previous studies, mice infected with mutant derivatives of *B. bronchiseptica* lacking the type III secretion system or adenylate cyclase toxin results in significantly less inflammation and respiratory histopathology [35], [36]. PMN may enhance shedding by causing inflammation that aggravates processes like coughing and sneezing. Products of inflammation sensitize sensory-afferent C-fibers of the bronchopulmonary tree, decreasing the threshold for activation of cough reflexes (21). Additionally, mucous production, sinusitis, post nasal drip and sneezing are associated with inflammation of the respiratory mucosa [37]–[39]. Mice are not known to cough, but post infectious nasal histopathology and efflux of mucous from the nasal cavity has been shown to be dependent on recruitment of PMN [40]–[42]. The association of PMN with increased shedding suggests that *Bordetella* may have evolved to induce neutrophil responses in order to enhance their transmissibility. Future work investigating histopathology occurring in the nares may help to describe how PMN recruitment and bacterial virulence factor expression relate to local tissue disruption, mucous secretion and shedding.

We observed that innate immunity, but not adaptive immunity is central to the control of *B. bronchiseptica* transmission; however, vaccines seek to prevent infection by inducing B and T cell responses. Although adaptive immunity did not affect growth and shedding of *B. bronchiseptica* in our model, they may play a more substantial role in a secondary bacterial challenge. In addition to direct antimicrobial effects, primed B and T cells may enhance innate immune responses [43], [44]. The ability of primed lymphocytes to prevent transmission may also depend on their anatomical residence. We observed that despite significant exposure to infectious organisms, WT mice did not become colonized, suggesting that innate defenses at the mucosal surface are central to host resistance or susceptibility to initial colonization (Figure 6). In contrast to parenteral vaccines, mucosal vaccines induce expression of site specific integrins that target lymphocytes to the site of induction. Mucosal vaccinations may therefore enable more immediate and robust responses to pathogens as they seed mucosal tissues [45].

Although vaccination protects the majority of individuals from severe disease, vaccines do not effectively prevent transmission of *Bordetella* and thus fail to confer the full benefits of herd immunity. Incidence of *B. pertussis* has increased significantly over the past decades in the U.S.A. and other countries that maintain very high vaccine coverage, accounting for a significant proportion of all coughing illnesses in both vaccinated and unvaccinated individuals [46]–[51]. The WHO estimates that regardless of vaccination coverage the incidence of *B. pertussis* infection in children is nearly 100% by age fifteen (52) Failure of vaccines to prevent its transmission enables *B. pertussis* to remain a substantial burden of disease across the globe. With a model for quantifying transmission of a closely related pathogen, *B. bronchiseptica*, it will be possible to apply molecular approaches for manipulation of both the host immune system and bacterial virulence genes to resolve mechanistic interactions that directly affect transmission. In addition it will be possible to evaluate therapeutic interventions directly in the context of transmission. Modifications to vaccines that improve their ability to prevent transmission of *Bordetella* species are likely to be applicable to other pathogens and will not only have the potential to protect susceptible individuals from severe illness, but overall diminish the global burden of disease [52].

## Materials and Methods

### Mice

4-to 6-week old C3HeJ, C3HeOuJ as well as C57/BL6J mice were purchased from Jackson Laboratories (Bar Harbor ME) and held in our *Bordetella*-free, specific pathogen-free facility at The Pennsylvania State University. μMT, Rag2^−/−^ and TCRβδ^−/−^ mice obtained from Jackson Laboratories were bred and maintained in our facility. This study was carried out in strict accordance with the recommendations in the Guide for the Care and Use of Laboratory Animals of the National Institutes of Health. The protocol was approved by the Institutional Animal Care and Use Committee at The Pennsylvania State University at University Park, PA (#40029 Bordetella-host Interaction). All animals were anesthetized using isoflourane or euthanized using carbon dioxide inhalation to minimize animal suffering.

### Bacterial Strains

*Bordetella bronchiseptica* strain RB50 [53] was maintained on Bordet-Gengou agar (Difco) supplemented with 10% defibrinated sheep blood (Hema Resources) and 20 µg/ml streptomycin (Sigma-Aldrich). Liquid cultures were grown overnight in Stainer-Scholte broth at 37°C to midlog phase [54]

### Inoculation

Inocula were prepared from mid-log phase liquid cultures. Cultures were diluted to 2×10^4^ CFU/ml (100 CFU inocula) or 10^3^ CFU/ml (5 CFU inocula). Mice were anaesthetized with isofluorane, and a 5 µl droplet placed on nares to restrict inocula to the nares.

### Analysis of Leukocyte Recruitment

10 ml of PBS was perfused through the left ventricle of mice while venous runoff was collected from the orbit. Nasal bones were dissected and placed in 1 ml of DMEM (5% FBS + 1 mg/ml collagenase D (Roche)). Samples were incubated for 45 minutes at 37°C and disaggregated suspension over a 70 µm mesh screen. 2×10^6^ cells per well were added to 96 well plates. Samples were resuspended in 200∶1 anti-CD16/32 (BD Biosciences) in PBS + 2% FBS and incubated on ice for 20 minutes. Following wash, cells were incubated with the following antibodies in PBS + 2% FBS: (All antibodies purchased from BD Bioscience unless otherwise stated) anti-CD45:APC-cy7, anti-CD11b:Horizon V450, anti-Ly6G: APC (E Bioscience), anti-CD11c:Per-CP, Anti-F480:PE-Cy7 (E Bioscience) Anti I-A^d^:PE (E Bioscience), Anti-CD3:Horizon V450, anti-NK1.1:AF-700.

### Analysis of Leukocytosis

25 µl of blood was collected by capillary tube following puncture of the facial vein with a 21 gauge needle and placed in 10 µl of 6% EDTA solution pH8. Red blood cells were lysed by incubation for 5 minutes in 0.5 mL of 0.15 M Ammonium chloride, 10 mM bicarbonate, 0.1 mM bicarbonate solution at room temperature interrupted by addition of 0.5 mL of ice cold PBS. After wash, leukocytes were detected as described above.

### Quantification of Bacteria

Tissues were harvested, homogenized in 1 ml PBS and diluted for quantification on Bordet and Gengou agar (BG). Shedding was assessed by gentle swabbing the external nares for 10 seconds with a Dacron-polyester tipped swab. Swab tips were cut and placed into 1 ml of PBS. Samples were vortexed vigorously and cultured on BG agar.

### Depletion of Neutrophils

PMN were depleted by intraperitoneal injection of 0.5 mg of monoclonal antibody from the RB6-8C5 hybridoma [55] 24 hours prior to infection and every other day thereafter throughout the infectious course. Age matched control mice were given 0.5 mg polyclonal nonspecific IgG2b isotype antibody generated from the NCG2B.01.

### Statistical Analysis

Timecourse analysis was analyzed by fit to a generalized linear model GLM to determine the effect of group and day on outputs like within host colonization, blood PMN counts and shedding (Minitab v.16). The effect of group on colonization, blood PMN counts and shedding at individual time points was determined by ANOVA using a Tukey's simultaneous test for significance.

## Supporting Information

Figure S1 **Intense leukocyte and neutrophil recruitment to the nasal cavity is observed in HeJ but not HeN mice.** Representative images of the analysis of leukocytes recruited to the nasal cavity 7 days after challenge of HeN or HeJ mice with B. bronchiseptica. Total leukocytes (CD45+) (A) neutrophils (CD11b+/Ly6G+) (B) macrophages (F4/80+/MHCII+) (C) NK cells (NK1.1+) (D) B cells (B220+/MHCII+) (E) and T cells (CD3+) (F) recovered from the nasal cavity of mice immediately before (day 0) and 1, 3 and 7 days after inoculation of HeJ or HeN mice with 100 CFU of B. bronchiseptica.
(TIF)

Figure S2 **Macrophages and NK cells are recruited 3 days after to the nasal cavity 3 days after inoculation.** Symbols represent the mean ± standard error of flow cytometry-derived counts of white blood cell types: (A macrophages (F4/80+/MHCII+), (B) NK cells (NK1.1+), recovered from the nasal cavity of mice immediately before (day 0) and 1, 3 and 7 days after inoculation of HeJ (black diamonds) or HeN (white squares) mice with 100 CFU of B. bronchiseptica.
(TIFF)

Figure S3 **Shedding intensity correlates with neutrophil recruitment in both HeJ and HeN mice.** Shedding was detected by quantitative culture of bacteria obtained in a swab of the external nares. Blood was collected from a puncture of the facial vein immediately after swab. CFU of B. bronchiseptica shed from individual HeN (white squares) or HeJ (black diamonds) mice swabbed at 7 days after inoculation and plotted as a function of blood neutrophil counts derived by flow cytometric detection of CD45+/CD11b+ and Ly6G+ cells.
(TIFF)

Figure S4 **7 Day treatment with anti-GR1 antibody successfully depletes neutrophils.** HeN mice were given intraperitoneal injection of 0.5 mg of either isotype control IgG2b (A) or anti-GR1 antibody (B). HeJ mice were given an intraperitoneal injection of 0.5 mg of either isotype control IgG2b (C) or anti-GR1 antibody (D). Flow cytometry plots represent the distribution of CD11b +/Ly6G+ cells in single cell suspensions recoveredd from mouse nasal cavities 7 days after they were inoculated with *B. bronchiseptica* and treated with antibodies. of HeJ (black diamonds) or HeN (white squares).
(TIFF)

Figure S5 **Prolonged anti-GR1 treatment results in enhanced neutrophil recruitment and inceased shedding in HeN but not HeJ mice.** (A) HeN or (D) HeJ mice were given 0.5 mg IP of isotype control IgG2b (HeN white square; HeJ black diamond) or anti-GR1 (black crosses) antibodies one day prior to infection and every other day thereafter for 14 days. Shedding was detected by culture of bacteria obtained swab of the external nares. Symbols represent the mean log10 CFU of B. bronchiseptica ± SEM. 14 days after inoculation, nasal neutrophil counts were derived from flow cytometric detection of CD45+/CD11b+ and Ly6G+ cells present in (B) control HeN (white bars) and GR1-treated HeN (striped bars) mice or (E) control HeJ (black bars) and GR1-treated HeJ (striped bars) mice. Bars represent the mean number of neutrophils ± SEM. CFU of B. bronchiseptica recovered from the nasal cavity of (C) HeN or (F) HeJ mice treated with either isotype control IgG2b (HeN white bars; HeJ black bars) or anti-GR1 antibodies (striped bars) 14 days after inoculation. Bars represent the mean log10 CFU of B. bronchiseptica ± the standard error. Dashed line represents the limit of detection.
(TIFF)
