# Supplementary figures and images for: Toll-Like Receptor 4 Limits Transmission of Bordetella bronchiseptica
Source: PLoS One. 2014 Jan 30;9(1):e85229. doi: 10.1371/journal.pone.0085229 (PMC3907416; doi:10.1371/journal.pone.0085229)

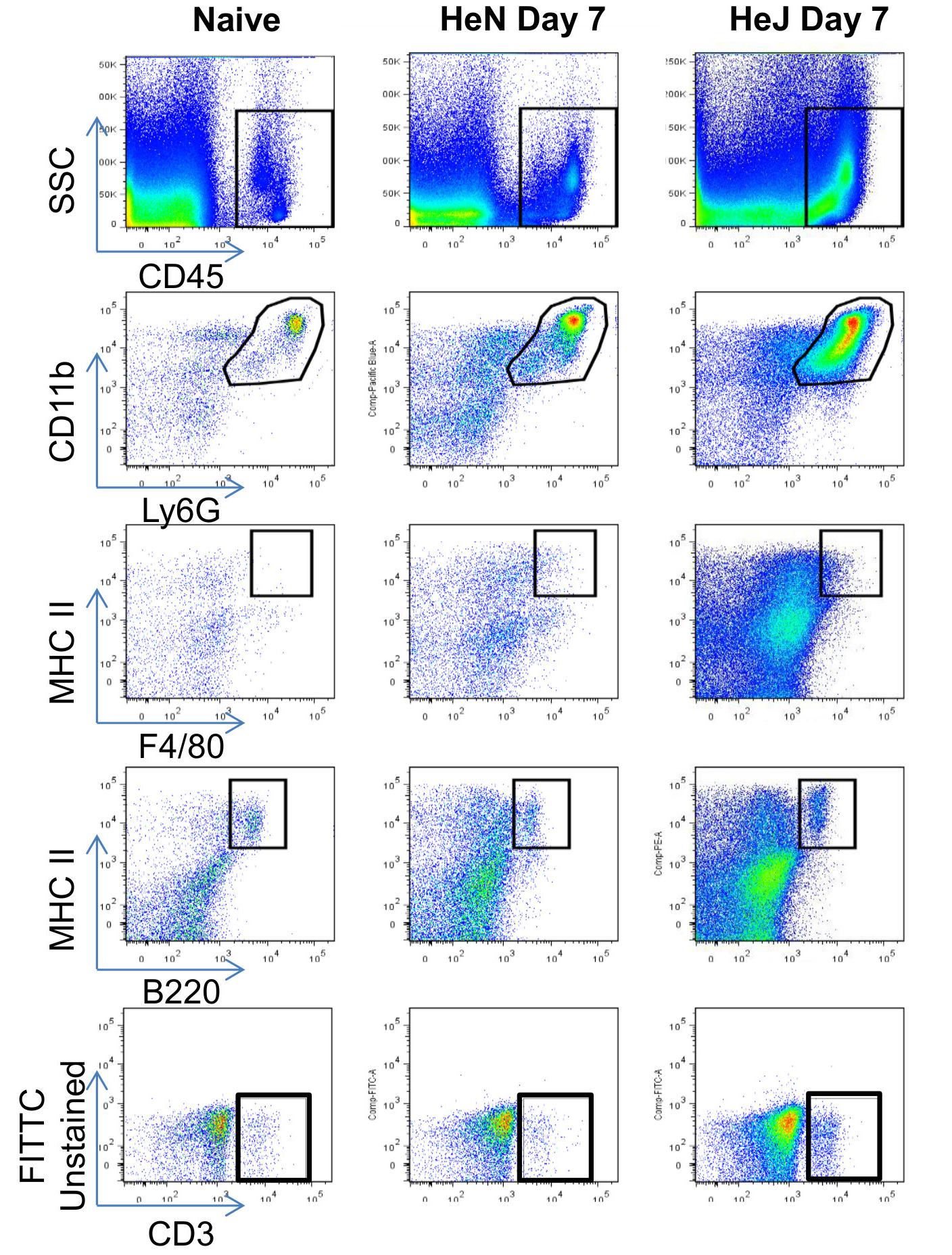

Supplement: Figure S1 — Intense leukocyte and neutrophil recruitment to the nasal cavity is observed in HeJ but not HeN mice. Representative images of the analysis of leukocytes recruited to the nasal cavity 7 days after challenge of HeN or HeJ mice with B. bronchiseptica. Total leukocytes (CD45+) (A) neutrophils (CD11b+/Ly6G+) (B) macrophages (F4/80+/MHCII+) (C) NK cells (NK1.1+) (D) B cells (B220+/MHCII+) (E) and T cells (CD3+) (F) recovered from the nasal cavity of mice immediately before (day 0) and 1, 3 and 7 days after inoculation of HeJ or HeN mice with 100 CFU of B. bronchiseptica. (TIF) [file pone.0085229.s001.tif]

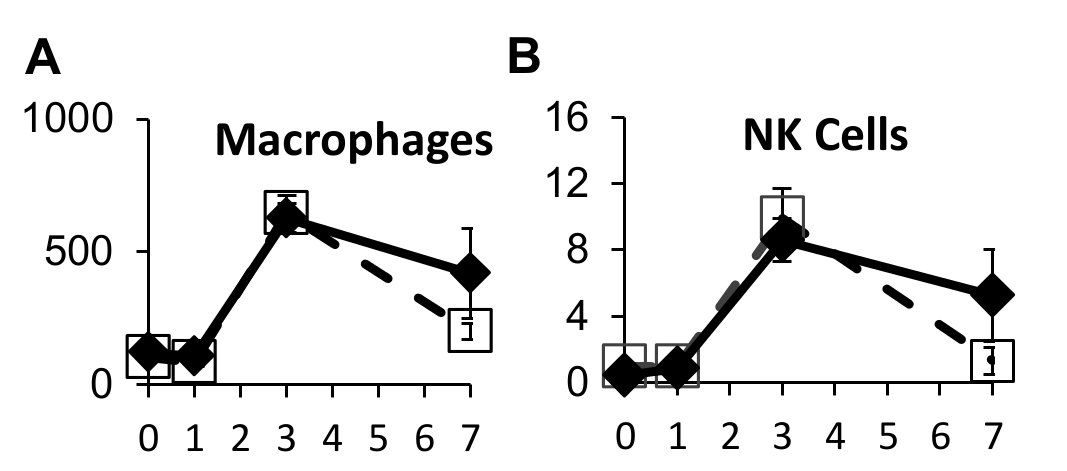

Supplement: Figure S2 — Macrophages and NK cells are recruited 3 days after to the nasal cavity 3 days after inoculation. Symbols represent the mean ± standard error of flow cytometry-derived counts of white blood cell types: (A macrophages (F4/80+/MHCII+), (B) NK cells (NK1.1+), recovered from the nasal cavity of mice immediately before (day 0) and 1, 3 and 7 days after inoculation of HeJ (black diamonds) or HeN (white squares) mice with 100 CFU of B. bronchiseptica. (TIFF) [file pone.0085229.s002.tiff]

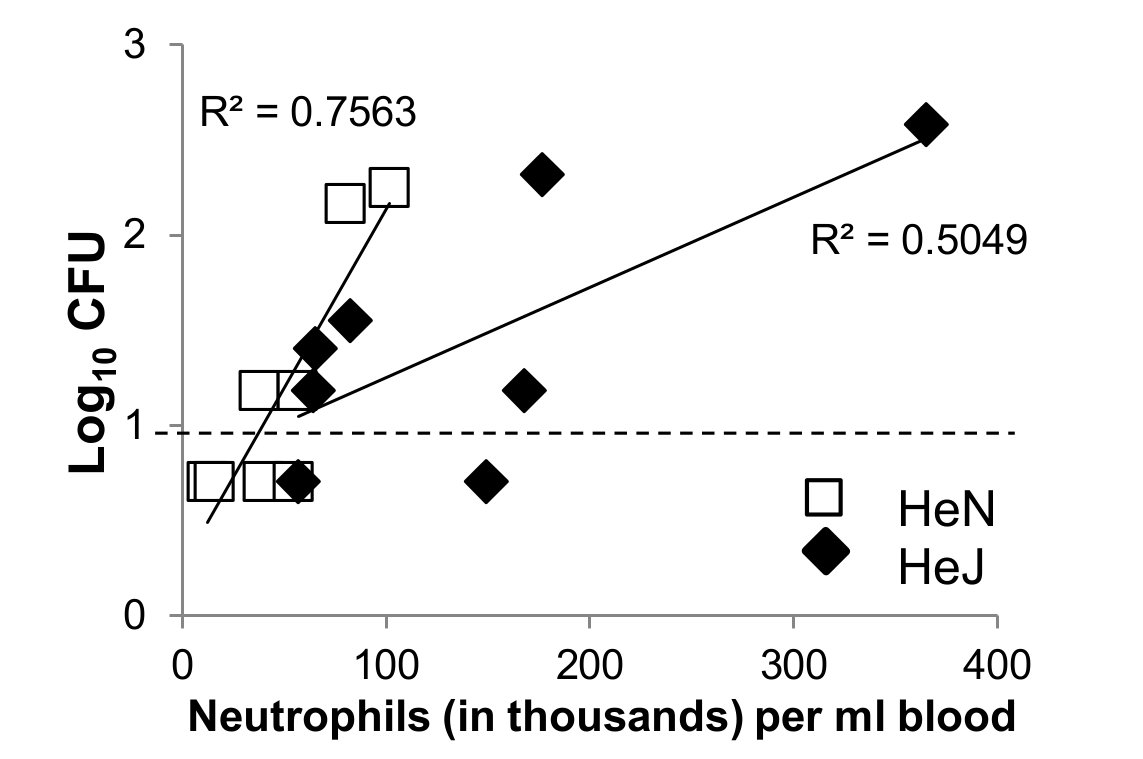

Supplement: Figure S3 — Shedding intensity correlates with neutrophil recruitment in both HeJ and HeN mice. Shedding was detected by quantitative culture of bacteria obtained in a swab of the external nares. Blood was collected from a puncture of the facial vein immediately after swab. CFU of B. bronchiseptica shed from individual HeN (white squares) or HeJ (black diamonds) mice swabbed at 7 days after inoculation and plotted as a function of blood neutrophil counts derived by flow cytometric detection of CD45+/CD11b+ and Ly6G+ cells. (TIFF) [file pone.0085229.s003.tiff]

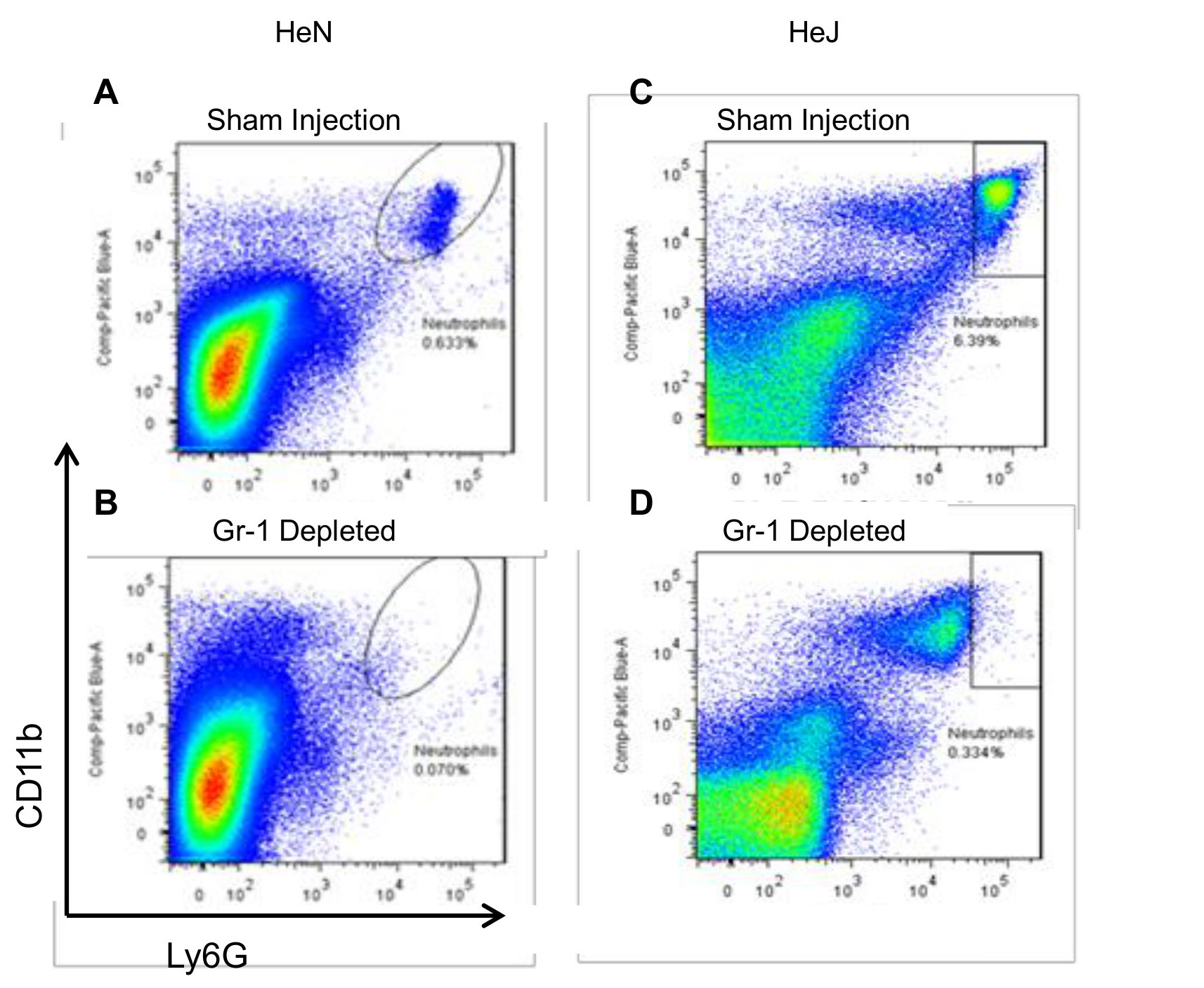

Supplement: Figure S4 — 7 Day treatment with anti-GR1 antibody successfully depletes neutrophils. HeN mice were given intraperitoneal injection of 0.5 mg of either isotype control IgG2b (A) or anti-GR1 antibody (B). HeJ mice were given an intraperitoneal injection of 0.5 mg of either isotype control IgG2b (C) or anti-GR1 antibody (D). Flow cytometry plots represent the distribution of CD11b +/Ly6G+ cells in single cell suspensions recoveredd from mouse nasal cavities 7 days after they were inoculated with B. bronchiseptica and treated with antibodies. of HeJ (black diamonds) or HeN (white squares). (TIFF) [file pone.0085229.s004.tiff]

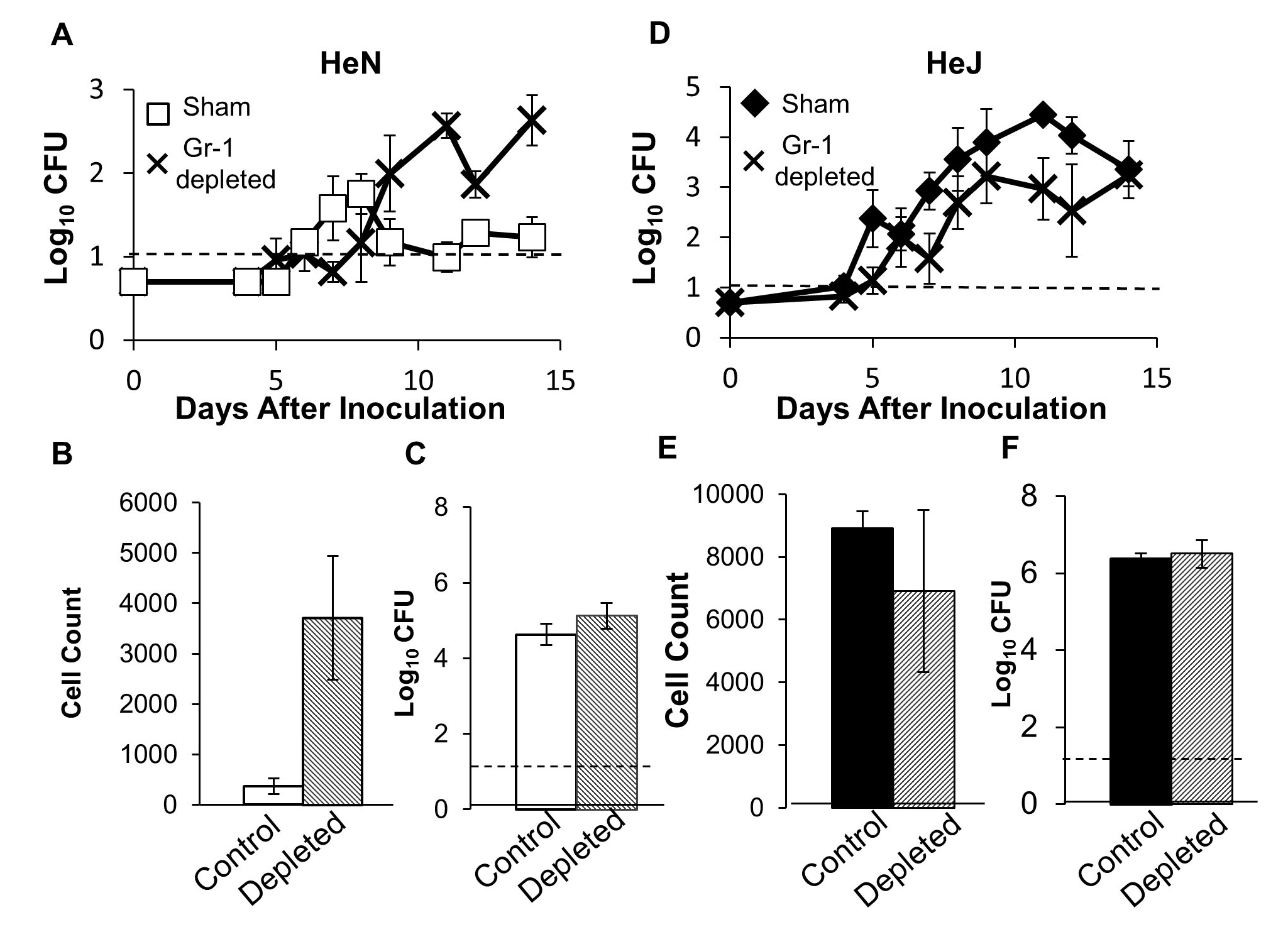

Supplement: Figure S5 — Prolonged anti-GR1 treatment results in enhanced neutrophil recruitment and inceased shedding in HeN but not HeJ mice. (A) HeN or (D) HeJ mice were given 0.5 mg IP of isotype control IgG2b (HeN white square; HeJ black diamond) or anti-GR1 (black crosses) antibodies one day prior to infection and every other day thereafter for 14 days. Shedding was detected by culture of bacteria obtained swab of the external nares. Symbols represent the mean log10 CFU of B. bronchiseptica ± SEM. 14 days after inoculation, nasal neutrophil counts were derived from flow cytometric detection of CD45+/CD11b+ and Ly6G+ cells present in (B) control HeN (white bars) and GR1-treated HeN (striped bars) mice or (E) control HeJ (black bars) and GR1-treated HeJ (striped bars) mice. Bars represent the mean number of neutrophils ± SEM. CFU of B. bronchiseptica recovered from the nasal cavity of (C) HeN or (F) HeJ mice treated with either isotype control IgG2b (HeN white bars; HeJ black bars) or anti-GR1 antibodies (striped bars) 14 days after inoculation. Bars represent the mean log10 CFU of B. bronchiseptica ± the standard error. Dashed line represents the limit of detection. (TIFF) [file pone.0085229.s005.tiff]
